# Southern Branch, National Dental Association, Second Annual Meeting, New Orleans, La., February 9, 10, 11 and 13, 1899

**Published:** 1899-07

**Authors:** Jennie M. Walker

**Affiliations:** Christian Pass, Miss.


### PROCEEDINGS.

### Southern Branch, National Dental Association, Second Annual Meeting, New Orleans, La., February 9, 10, 11 and 13, 1899.

Reported for the DENTAL REGISTER by Mrs. JENNIE M. WALKER, Pass Christian, Miss.

[*Continued from June No., Page* 279.]

### MICROSCOPIC EFFECTS OF CATAPHORESIS.

BY A. F. SONNTAG, WACO, TEX.

Abstract of paper read.

This interesting paper was illustrated by lantern slides, showing the penetration into dentinal tubuli of colored cocain solutions, used by the cataphoric process. It was shown very clearly that the solution on cotton placed in the cavity, without the aid of the current, does not penetrate at all. Also that, even with the current, it does not penetrate the layer of carious dentin. It is necessary to remove the carious dentin at least from one point for the solution to penetrate the dentin. In one case the cotton saturated with the colored cocain solution was placed in the cavity without removal of the carious dentin; a wisp of the cotton was allowed to reach over to a sound portion of the tooth from which the enamel had been purposely removed. The electrode was placed on the cotton in the cavity and the current applied. There was no penetration of the colored fluid in the cavity, but from the spot whence the enamel had been removed the fluid penetrated the dentinal tubules quite distinctly, the current having followed the wisp of cotton, taking the longer route toward the pulp, the carious dentin in the cavity opposing no barrier to its passage.

### ORAL HYGIENE.

BY DR J. P. CORLEY, GREENSBORO, ALA.

He discussed the question whether immunity from dental caries can best be accomplished through local or systemic measures. If caries is of local origin, then our domain is restricted to the mouth, and we can never hope to be more than therapeutists. If, on the other hand, it is due solely to systemic pathology, we should be dental physicians. But if the pathogenesis of dental caries is both local and systemic, then the province of the dental profession is co-extensive with that of general medicine. Dentistry and medicine should co-operate in the endeavor to secure sound teeth to future generations, and dentistry should have for its highest aim the prevention of caries. Quoting from Dr. J. Leon Williams, to the effect that acid-forming bacteria are the sole active cause of dental caries and the predisposing cause to be found in that "peculiar condition of the bodily juices and cells, in which they are unable to repel the invasion of pathological micro-organisms," and the causes of caries thus both local and general. Dr. Corley next discussed the question as to which influence was the most potent. As against the statement that "the mouth affords an ideal hotbed for the culture and propagation of a hundred different kinds of pathological micro-organisms," he cites the facts that wounds in the mouth heal more readily than elsewhere, that animals always bathe their wounds in saliva, using the tongue to cleanse and disinfect; that the distinctive acid product of the micro-organisms is being continually diluted with hundreds of times its own volume of alkaline fluids from the mucous and salivary glands. Summing up, the author bases his theory of immunity on the trio—dietetic, physiologic and psychologic influences. Food should be such as to afford teeth, gums, mucous membranes and muscles of mastication abundant exercise, and should be taken leisurely and with mental relaxation; drinks should be free from stimulants and of a temperature such as not to interfere with the action of the ptyalin of the saliva.

Physical auxilliaries, such as exercise, baths, ventilation, are essential. As to psychological influences, a clear conscience has a wonderful influence on digestion, nutrition, assimilation and metabolism, and normal metabolism gives immunity from caries.

DISCUSSION.

DR. COWARDIN does not consider vitality an important factor in the question of dental caries, or that the teeth have within themselves any inherent power of repelling decay. Teeth from which the pulps have been removed do not decay any more rapidly than those in which the pulp is performing all its functions.

DR. J. Y. CRAWFORD spoke of the care required by the first permanent molars. As soon as the crown of the teeth are laid bare they should be washed and made perfectly clean—made aseptic—and the occlusal surface covered with a good cement, so that all imperfections may be perfectly sealed. If the cement washes out, it should be renewed as often as necessary until they have passed the period of susceptibility and become immune to caries, with perfect grinding surfaces. These teeth are made up at a period of life which renders them peculiarly susceptible to caries, but under the protecting cement fissures calcify and the surface hardens. What was a virgin field for the implantation of disease germs becomes immune if properly protected.

In closing the discussion Dr. Corley said, in reply to Dr. Cowardin, that the gentleman apparently overlooked the fact that the teeth are still possessed of vitality after the removal of the pulp, and may render good service for years; but when, from other causes than the loss of the pulp, the tooth becomes really a *dead tooth*, then it is soon cast off. Nature will not tolerate dead tissue.

### SECTIONAL BRIDGEWORK.

BY DR. E. P. BEADLES.

Abstract of paper read.

It is generally recognized that bridges should be as short as possible. A greater strength is obtained; in case of breakage repair can be more easily made, and short bridges are more easily cemented on. Dr. Beadles described the following case illustrative of these principles: The anchorages consisted of the superior centrals, left lateral, second bicuspids, right and left, and second molars, right and left. The first bridge made and cemented on extended from left second molar to second bicuspid, each of which carried a shell crown. The bicuspid crown was made thin and without cuspids. For the second bridge the left lateral root was fitted with pin and cap and a telescoping crown made to cover the previously crowned bicuspid, with a slot in the side to make room for the soldered attachment of the bridge already in place. The space from left lateral to second molar was thus filled with two bridges. The roots of the centrals were next prepared with pins and caps, with a shell crown without cusps for the second right bicuspid. This third bridge, which carried the intervening right lateral, right cuspid and and first right bicuspid, was then cemented to place. A telescoping cusp-crown was then made for the second bicuspid and a crown for the second molar, thus filling the upper arch with four bridges, one anchorage tooth on either side bearing the ends of two bridges, one telescoped over the other. This method has been employed by Dr. Beadles in several cases, with great satisfaction.

### PLAIN RUBBER TEETH IN BRIDGEWORK.

BY DR. WM. H. COOKE.

Dr. Cooke suggests the use of plain rubber teeth in bridgework, in place of facings or veneers, for molars and bicuspids. With a diamond disk a shoulder is cut below the pins and a thin backing of gold burnished to the back and shoulders, extending to the masticating surface and beyond the apex of the gum portion, where it is burnished over and around the end, forming a cup in which the tooth rests. Flow sufficient solder over the backing to secure the necessary strength for mastication.

### CLINICS.

Preceding the clinics Drs. L. M. Cowardin and H. Stuart

MacLean, of Richmond, Va., displayed a valuable collection of microscopic slides prepared by Sam G. P. Cowardin, F. R. M. S. A number of high-power microscopes were arranged upon a large table so that those most interested in this study had an opportunity of examining numerous slides, representing both normal and pathologic conditions of tooth tissues and general histologic and pathologic tissues and bacteriologic preparations.

Dr. T. C. West, of Natchez, Miss., demonstrated his improved methods in plate-work.

Dr. H. H Johnson, of Macon, Ga., showed a new method of strengthening a decayed root for crown attachment. Dr. Johnson exhibited, among other anomalies and dental curios, two sets of teeth belonging to the same patient who had actually *worn out* the porcelain teeth ; one set being ground down to the base, the other to the level of the platinum pins, which had cut grooves in the opposing teeth. Dr. Johnson also showed a cast of a jaw in which the second bicuspid had erupted posterior to the first permanent molar. Also casts of the jaws of a man forty years of age in which nearly all of the deciduous teeth, and also the permanent teeth were in position in the lower jaw, a number of deciduous teeth being present in the upper jaw also.

Dr Merchant stated that Dr. Staples had presented the museum of the Texas Dental Society with casts of almost a parallel case.

Dr. Crawford said that he had seen the casts of the latter case and had supposed that Dr. Johnson's were duplicates of the same case. This is not the case, however.

Dr. C. L. Alexander, Charlotte, N. C., demonstrated a new method of retention of rubber plates, adaptable to any difficult cases. He also demonstrated his method of making "cast fillings."

Dr. George B. Clement, Macon, Miss., demonstrated his new alveolar amputating forceps, designed for the immediate trimming of the alveolar ridge, removing all projecting points of socket walls and septa, placing the ridge at once in the rounded smooth condition to which nature brings it by the slow, tedious process of absorption, facilitating the healing process of the soft tissues and putting the mouth in condition for reception of artificial dentures in the course of a few weeks, instead of months. In this clinic, the remains of fourteen teeth in the upper jaw were removed, and the alveolar ridge smoothly trimmed in about four minutes without the use of anesthetics. Dr. Clement's forceps has long, flat beaks which are to be pressed firmly down between the alveolar wall and periosteum, the latter being carefully preserved for the part it plays in the healing process. In the extraction of roots flat beak serves as a universal elevator. Dr. Clement claims that the process of trimming is effected without additional shock to the patient if performed immediately; that it lessens subsequent pain, soreness and discomfort, and greatly shortens the time between extraction of teeth and insertion of substitutes.

In the discussion of this clinic, Dr. J. Rollo Knapp expressed the opinion that the patient had been subjected to undue suffering, and that the final result would not be found to justify the operation.

On motion of the Chairman of Clinics, Dr. T. P. Hinman, a committee of three local dentists, Drs. Knapp, Bauer and Vignes, was appointed to watch the case and report upon the condition of the mouth at the expiration of a month from the date of operation.

Dr. J. Y. Crawford commended the operation as based upon the profoundest principles of oral surgery and as exemplifying the rule laid down by "the grand old Atkinson" in the preparation of a mouth for the reception of artificial teeth, the immediate surgical removal of all that nature would otherwise remove by a tedious and painful process. The jagged margins of the alveolar process, and the numerous septa, causes suffering, prolonged sometimes for weeks and months, as the scar-tissue of the healing soft tissues is drawn taut across the projecting sharp points of bone. It is a humane operation to present, by means of a surgical operation, to the torn and mangled gums a smoothly trimmed, rounded surface, over which they heal rapidly by first intention.

Dr. C. R. Vignes, New Orleans, demonstrated the treatment of pulpless teeth with iodoform vapor under pressure, using the Blair Vaporizer.

Dr. T. C. West said that his patients objected so strenuously to the odor of iodoform that he had been led to experiment with other drugs in the vaporizer, and had for several years been obtaining uniformly successful results by the substitution of acetanilid for iodoform.

Dr. A. J. Foret, New Orleans, demonstrated the use of the electric helix for obtunding sensitive dentin.

Dr. T. P. Hinman, Atlanta, Ga., made a porcelain inlay with the Jenkins' system, the case being the labial surface of a right superior central.

Dr. W. J. Younger, with a patient in the chair, described the methods he would employ in the correction of the irregularities presented by the use of silk ligatures. He laid particular stress upon the point that it is only a popular fallacy that silk thread tightens with moisture. Pure silk does not contract under moisture. The thread must be stretched until it will yield no more, before tying the knots—a surgeon's knot, covered by a granny knot. It is the contractility of the silk thread, after being stretched, that gives it its wonderful power.

